# AI-based automated segmentation for ovarian/adnexal masses and their internal components on ultrasound imaging

**DOI:** 10.1117/1.JMI.11.4.044505

**Published:** 2024-08-06

**Authors:** Heather M. Whitney, Roni Yoeli-Bik, Jacques S. Abramowicz, Li Lan, Hui Li, Ryan E. Longman, Ernst Lengyel, Maryellen L. Giger

**Affiliations:** a The University of Chicago, Department of Radiology, Chicago, Illinois, United States; b The University of Chicago, Department of Obstetrics and Gynecology/Section of Gynecologic Oncology, Chicago, Illinois, United States; c The University of Chicago, Department of Obstetrics and Gynecology/Section of Ultrasound, Genetics, and Fetal Neonatal Care Center, Chicago, Illinois, United States

**Keywords:** adnexal diseases, ovarian cancer, ultrasound, segmentation, machine learning, deep learning

## Abstract

**Purpose:**

Segmentation of ovarian/adnexal masses from surrounding tissue on ultrasound images is a challenging task. The separation of masses into different components may also be important for radiomic feature extraction. Our study aimed to develop an artificial intelligence-based automatic segmentation method for transvaginal ultrasound images that (1) outlines the exterior boundary of adnexal masses and (2) separates internal components.

**Approach:**

A retrospective ultrasound imaging database of adnexal masses was reviewed for exclusion criteria at the patient, mass, and image levels, with one image per mass. The resulting 54 adnexal masses (36 benign/18 malignant) from 53 patients were separated by patient into training (26 benign/12 malignant) and independent test (10 benign/6 malignant) sets. U-net segmentation performance on test images compared to expert detailed outlines was measured using the Dice similarity coefficient (DSC) and the ratio of the Hausdorff distance to the effective diameter of the outline ($R_{\mathrm{HD}\text{-}D}$) for each mass. Subsequently, in discovery mode, a two-level fuzzy c-means (FCM) unsupervised clustering approach was used to separate the pixels within masses belonging to hypoechoic or hyperechoic components.

**Results:**

The DSC (median [95% confidence interval]) was 0.91 [0.78, 0.96], and $R_{\mathrm{HD}\text{-}D}$ was 0.04 [0.01, 0.12], indicating strong agreement with expert outlines. Clinical review of the internal separation of masses into echogenic components demonstrated a strong association with mass characteristics.

**Conclusion:**

A combined U-net and FCM algorithm for automatic segmentation of adnexal masses and their internal components achieved excellent results compared with expert outlines and review, supporting future radiomic feature-based classification of the masses by components.

## 1 Introduction

Adnexal masses can be found in the ovary, fallopian tube, or surrounding tissue and represent a heterogeneous spectrum of benign, borderline, and malignant entities.^1^ They are common, with an incidence of 35% in premenopausal and 17% in postmenopausal women.^2^ Pathologies differ across age ranges and geographical areas,^1^^,^^3^^,^^4^ but most adnexal masses (∼85%) are benign and without symptoms. Most can be managed conservatively with a follow-up by clinical exam and sequential imaging, without surgical intervention.^5^^–^^7^ It is estimated that 10% of all women will be operated on for an adnexal mass during their lifetime,^7^ potentially resulting in peri- and postoperative morbidity (e.g., infections, injury to adjacent organs, and anesthetic complications). However, although most adnexal masses are benign, and ovarian cancer is a rare disease with an incidence of one case per 91 women, it is the most lethal gynecology malignancy, with a 5-year survival rate of only 30% when diagnosed in advanced stages.^8^^,^^9^

Ultrasound imaging plays a key role in the evaluation of patients with adnexal masses.^6^ It is noninvasive, widely available, safe, and low cost. Assessments are based mainly on qualitative features, including mass morphology, margins and echogenicity, the presence of solid elements, acoustic shadowing, vascular flow signals, and interaction with the surrounding tissues.^10^ Given that ultrasound imaging is used early during the evaluation of any lower abdominal, back, or flank pain,^11^ the incidental detection of adnexal masses has substantially increased. Several ultrasound-based risk models have been developed to standardize adnexal mass assessment, such as the International Ovarian Tumor Analysis (IOTA) Simple Rules,^12^ the IOTA Assessment of Different NEoplasias in the adneXa model,^13^ and the American College of Radiology Ovarian-Adnexal Reporting and Data (O-RADS)^14^ risk stratification system with the goal of reducing false positive and false negative assessments. Recent studies comparing the performance of the risk stratification systems in differentiating between benign and malignant adnexal masses in cohorts in the United States reported strong performance of all models.^15^^,^^16^ However, these models rely on qualitative assessments of the masses, which are subject to inter- and intra-observer variability,^17^ and achieving expertise in ultrasound interpretation takes time to gain and is not ubiquitously available.^6^^,^^18^^,^^19^

Artificial intelligence (AI)-based automation for the assessment of adnexal masses on ultrasound images may provide decision support tools that are more quantitative, more robust, and better performing than qualitative-based systems, similar to other advances in oncology, such as breast cancer,^20^^–^^22^ lung cancer,^23^ and melanoma.^24^ Our long-term goal is to develop an AI-based pipeline that improves diagnostic accuracy for adnexal masses and decreases unnecessary surgeries for asymptomatic benign masses while being efficiently integrated into a clinical workflow. The first step is to automatically (i.e., objectively) outline the area of interest, the adnexal mass. This segmentation task includes identifying the extent of the abnormal tissue and distinguishing it from the surrounding tissue. When done manually, segmentations require expertise, are time-consuming, and are prone to errors even when done by experts.^25^^,^^26^ Moreover, because adnexal masses are heterogeneous, a reproducible system that separates the internal components by echogenicity will enable additional radiomic analysis by components, such as size and shape.

The purpose of this study was to develop an automated two-step segmentation technique for adnexal masses using (1) a supervised deep learning (DL) algorithm to automatically segment the masses from surrounding tissue and (2) an unsupervised algorithm to automatically separate the interior parts of the masses by echogenic components.

## 2 Methods

### 2.1 Dataset

The research used a retrospectively collected, deidentified dataset from a previously described database, including clinical information and ultrasound images of more than 500 consecutive patients undergoing evaluation for an adnexal mass in the Department of Obstetrics and Gynecology at the University of Chicago Medical Center in the Section of Ultrasound, Genetics, and Fetal Neonatal Care Center.^16^ The data had been collected under an IRB-approved protocol, with all images having been acquired using either GE Voluson E8 or E10 or Samsung Elite WS80 ultrasound systems between January 2017 and June 2023. Borderline masses were considered malignant for the purposes of this study, as they require surgery. Exclusion was conducted at the patient, adnexal mass, and image levels. Patients were excluded if they were managed conservatively (i.e., no surgery after imaging), if no follow-up information was available, or if the patient received imaging outside of the University of Chicago. Pelvic masses were excluded if they were not considered adnexal in origin. Images were excluded if the entire border of the mass was not visible, if the image had been acquired by transabdominal approach, or if the image had Doppler or measurement markups visible. Given the low prevalence of ovarian cancer, the dataset was also enriched by reviewing any contralateral malignant masses that were not excluded at the mass or image level. This resulted in the addition of three malignant masses into the dataset. There was a total of 133 patients with 136 unique adnexal masses in the dataset of images after the exclusion criteria and data enrichment were applied.

Note that this imaging dataset of 133 patients was collected for overall AI pipeline research and development, including both a segmentation stage and a classification stage. Thus it was prospectively and manually split into a classification training and validation (classification TV) set (a total of 95 patients) and an independent classification test set (41 patients). This split was conducted to balance the sets by mass pathology subtype and clinical parameters, such as menopausal status and race, and to ensure equal distribution for a future AI-based classification model.

**Fig. 1:**
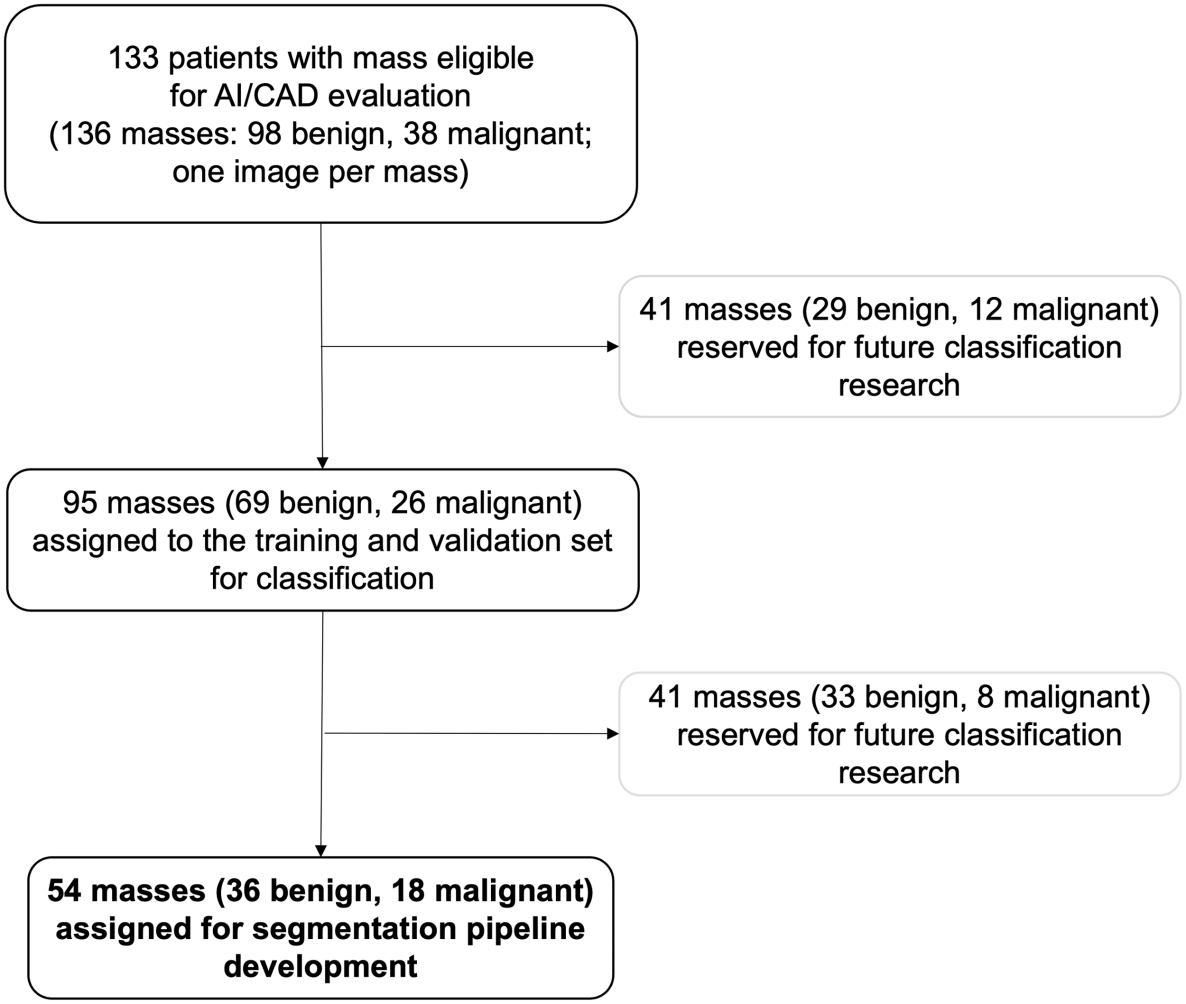
Consort diagram reporting the selection of adnexal masses used in the study. The final cohort for the U-net segmentation study was 54 unique masses (from 53 patients).

U-net segmentation techniques do not require a large number of masses for training, especially when training data is augmented, due to the operations being conducted at the pixel level.^27^^,^^28^ From the classification TV set, a subset of adnexal masses was chosen for the automated mass segmentation development. Thus, the resulting final dataset for the segmentation component of the pipeline development consisted of only 54 adnexal masses (36 benign and 18 malignant) from 53 unique patients with a median age of 43 years (range: 20 to 79 years) (Fig. 1) leaving 82 masses from 80 patients reserved for the future classification stage.

The final dataset of masses assigned for segmentation pipeline development was automatically split into a training set and a test set. The masses were separated by patient and by mass subtype into training (goal: 65%) and test (goal: 35%) for segmentation by stratified sampling^29^ (Table 1). This split proportion was chosen to ensure that each mass pathology subtype was included in the training and test sets whenever possible.

**Table 1:** Description of the adnexal mass dataset used for the segmentation pipeline development for adnexal masses. Percentages may not add to 100% due to rounding. Splitting was conducted at the patient level according to pathologist-reported findings.

Mass type	Pathology^a^	Training set		Test set	
		Masses (#)	Percent of the training set (%)	Masses (#)	Percent of test set (%)
Benign	Physiologic functional cyst or other miscellaneous mass^b^	5	13	1	6
	Endometrioma	5	13	2	13
	Epithelial origin mass	7	18	3	19
	Sex-cord stromal or germ cell mass	7	18	3	19
	Extra-ovarian benign pathology^c^	2	5	1	6
	Total	26	67	10	63
Malignant	Borderline ovarian tumor	1	3	1	6
	Epithelial invasive ovarian cancer	6	16	2	13
	Non-epithelial invasive ovarian cancer	4	11	1	6
	Secondary metastasis to the ovaries	1	3	2	13
	Total	12	33	6	37

a Pathologist reported findings.

b Includes denuded simple cysts, hemorrhagic cysts, and luteinized follicular cysts.

c Includes hydrosalpinx and periadnexal soft tissue lymphangioma.

### 2.2 Supervised Model for Segmentation of the Entire Adnexal Mass

#### 2.2.1 Model training

The borders of the adnexal masses were outlined by an experienced clinical researcher (RYB) followed by consensus from clinicians with more than 40 years of experience (JSA) and more than 20 years of experience (REL) in gynecological ultrasound interpretation. These consensus outlines were used as the reference to assess the segmentation of adnexal masses from surrounding tissue. Bounding boxes were placed around each mass, which served as the region of interest (ROI) for each mass.

Each image was cropped to the bounding box outline and resized to 256×256  pixels. The resized ROIs of the masses in the U-net training set were augmented using flips (left-right and up-down) and rotations (12 combinations total). For AI-based segmentation of the mass from the surrounding tissue, we used a U-net algorithm^30^ because of the large amount of data that the pixel-based method provides to the pipeline. The U-net model was trained to identify pixels within the resized expert ROI as either within or outside the mass. Parameters were set as follows: Adam optimizer, initial learning rate: 0.001; maximum epochs: 120; and minibatch size: 12 (MATLAB R2022b, MathWorks, Natwick, Massachusetts). The trained U-net was applied to the resized expert ROIs of the masses in the test set, resulting in a prediction mask.

**Fig. 2:**
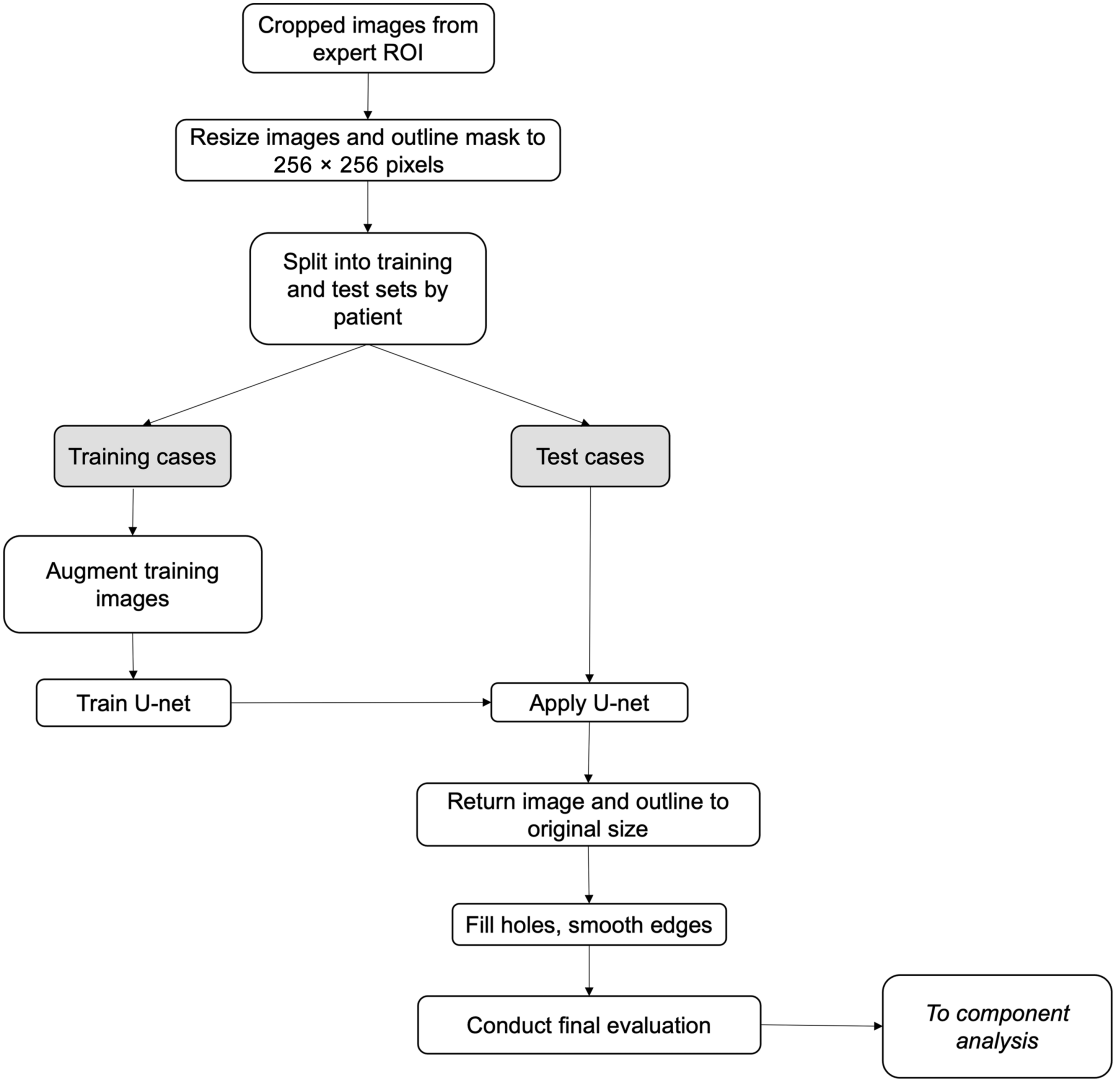
Workflow for supervised U-net segmentation of the adnexal mass.

For each image in the test set, the resized ROI that contained the U-net prediction of pixels within the mass was returned to the original size of the ROI. The prediction mask was filled in so that no holes were present. The boundary of the prediction mask was smoothed by applying a Gaussian kernel with a window size of 15 pixels to a two-dimensional convolution of the mask, and the final U-net prediction mask was set with a threshold of pixels >0.5. The final U-net prediction boundary for each mass was the outline of the boundary of the final U-net prediction mask for that image. Figure 2 shows the workflow for the U-net segmentation of masses.

#### 2.2.2 Model testing

The U-net performance on the masses in the test set was compared to the expert outlines using (1) the Dice similarity coefficient (DSC)^31^ and (2) $R_{\mathrm{HD}\text{-}D}$, defined as the ratio

$$R_{\mathrm{HD}\text{-}D}=\frac{\mathrm{HD}}{D_{\mathrm{eq}}},$$ (1)

where for each mass, HD is the average Hausdorff distance^32^^,^^33^ and $D_{\mathrm{eq}}$ is the effective diameter, i.e., the diameter of a circle with the same area as the region of the expert outline (Eq. 1). $R_{\mathrm{HD}\text{-}D}$ was useful as a dimensionless metric of the distance of the U-net prediction outline from the expert outline, consistent with the nature of the DSC.

### 2.3 Unsupervised Model for Segmentation of Internal Components

#### 2.3.1 Model development

**Fig. 3:**
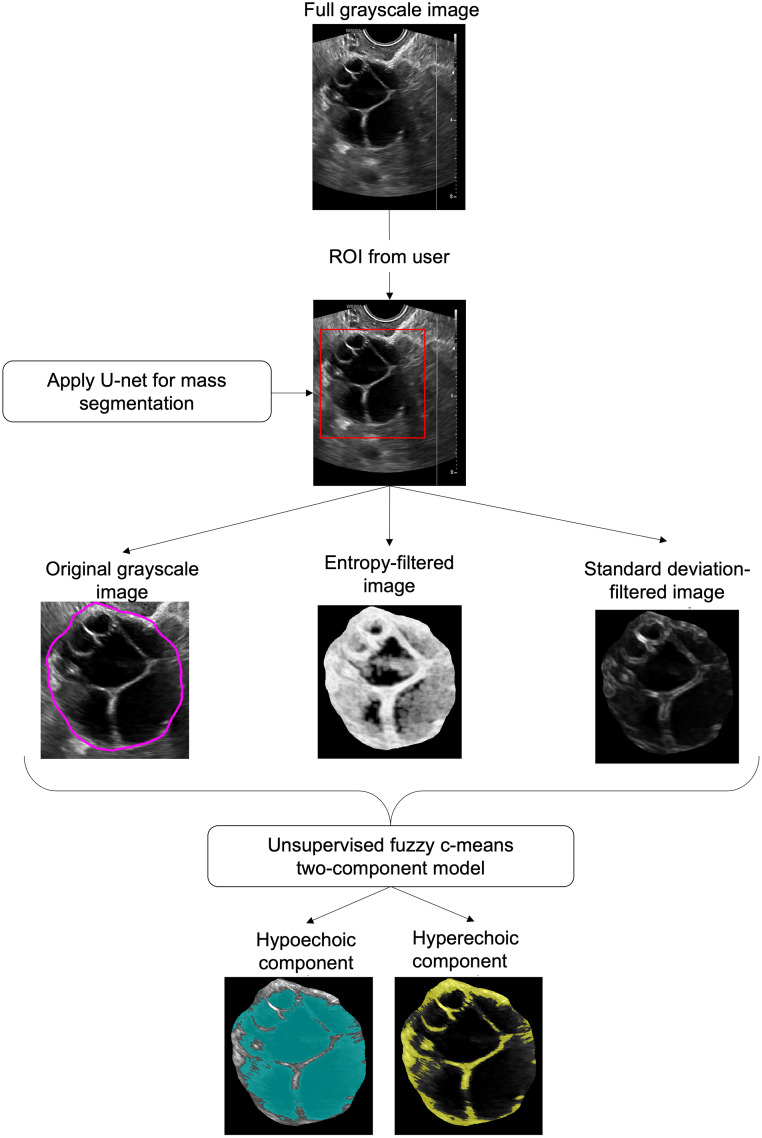
Workflow for segmentation of masses into internal components using an unsupervised FCM algorithm.

After the U-net described above was applied to each image, an unsupervised fuzzy c-means (FCM) algorithm^34^^–^^36^ was applied in a discovery mode to categorize pixels inside the segmented mass as belonging to one of the two components. The use of two components in the FCM was prospectively chosen due to the nature of most adnexal masses as containing low and high echogenic components. Three versions of the ultrasound image cropped to the expert bounding box outline were used as input to an unsupervised FCM algorithm: original grayscale, entropy-filtered, and standard-deviation filtered. These filtered versions were calculated using nine-pixel sliding neighborhoods of the entropy and standard deviation of each pixel. Entropy and standard deviation filters were chosen to emphasize the structure and magnitude, respectively, of the components within the mass. The mean of the pixels in each component was obtained, and the group of pixels with the lowest mean was identified as the relative “hypoechoic component.” From this, the other group of pixels was identified as the “hyperechoic component.” That is, hypoechoic pixels tended to have low grayscale, variation, and entropy, whereas hyperechoic pixels tended to have high grayscale, variation, and entropy. Figure 3 shows the workflow for identifying and segmenting the internal components of the masses.

#### 2.3.2 Clinical assessment

The associations of the hypoechoic and hyperechoic components in each image were reviewed by a clinical researcher (RYB) and expert clinician (JSA) for potential association with adnexal mass tissue properties, such as cystic, solid, or mixed components. This was conducted for the entire dataset.

## 3 Results

### 3.1 Supervised Model for Segmentation of the Entire Adnexal Mass

**Fig. 4:**
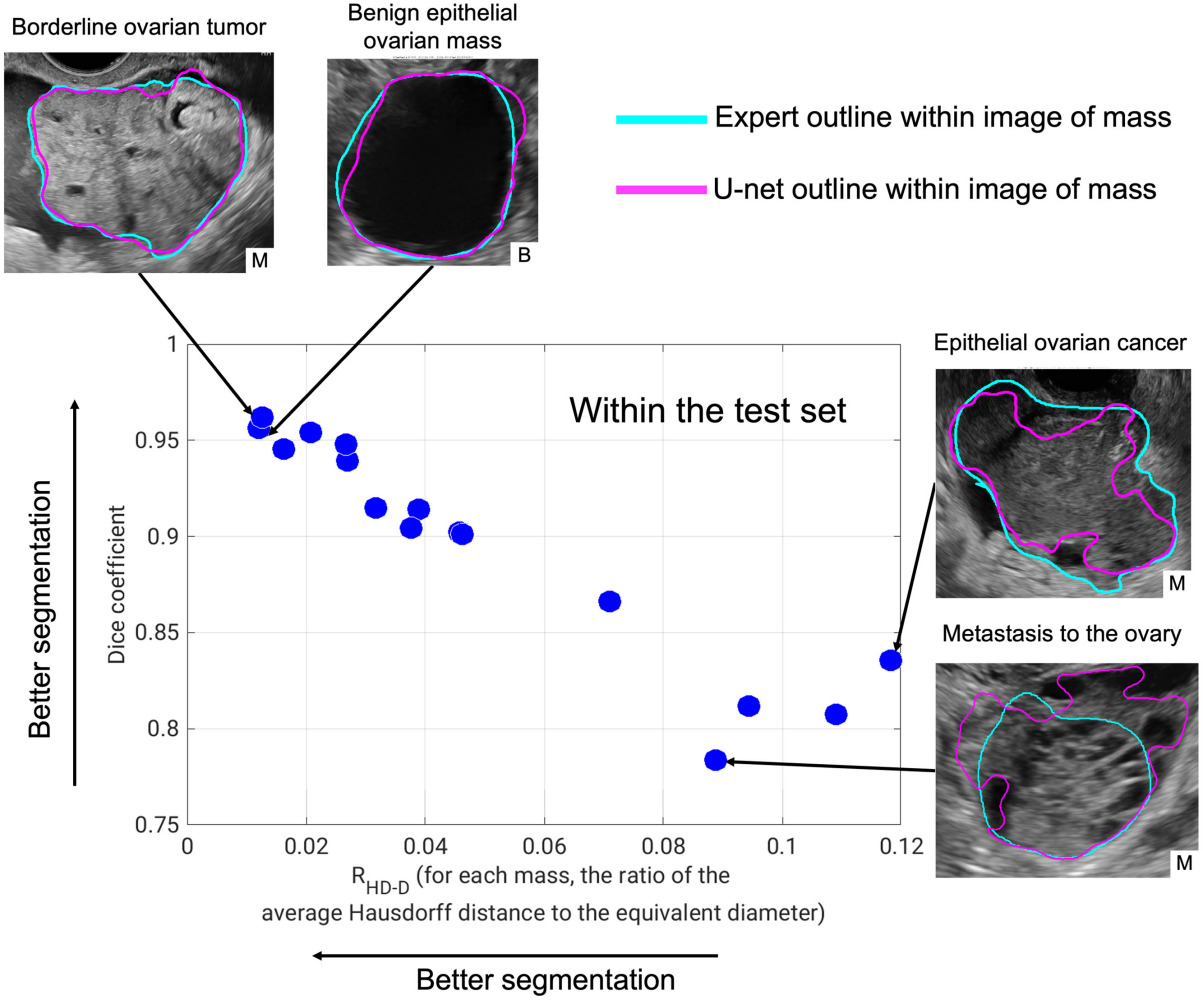
U-net segmentation performance in the test set in the task of segmenting the entire adnexal mass from the surrounding tissue, compared to expert outlines. ($R_{\mathrm{HD}\text{-}D}$: ratio of the average Hausdorff distance to the effective diameter of the mass.) Images of the four masses with the best performance (highest Dice coefficient and lowest $R_{\mathrm{HD}\text{-}D}$) and lowest performance (lowest Dice coefficient and highest $R_{\mathrm{HD}\text{-}D}$) are shown. Clockwise from top left with pathology (with patient diagnosis) details: borderline ovarian mass (borderline serous tumor), epithelial ovarian mass (benign serous cystadenoma), epithelial ovarian cancer (high-grade serous ovarian cancer), and metastasis to the ovaries (cancer of gastro-intestinal primary origin). B, benign and M, malignant.

The Dice coefficient (median [95% confidence interval]) was 0.91 [0.78, 0.96] and $R_{\mathrm{HD}\text{-}D}$ was 0.04 [0.01, 0.12] in the test set (Fig. 4), indicating strong performance of the U-net in the task of segmenting adnexal masses from the surrounding tissue compared to expert outlines.

### 3.2 Unsupervised Model for Segmentation of Internal Components

**Fig. 5:**
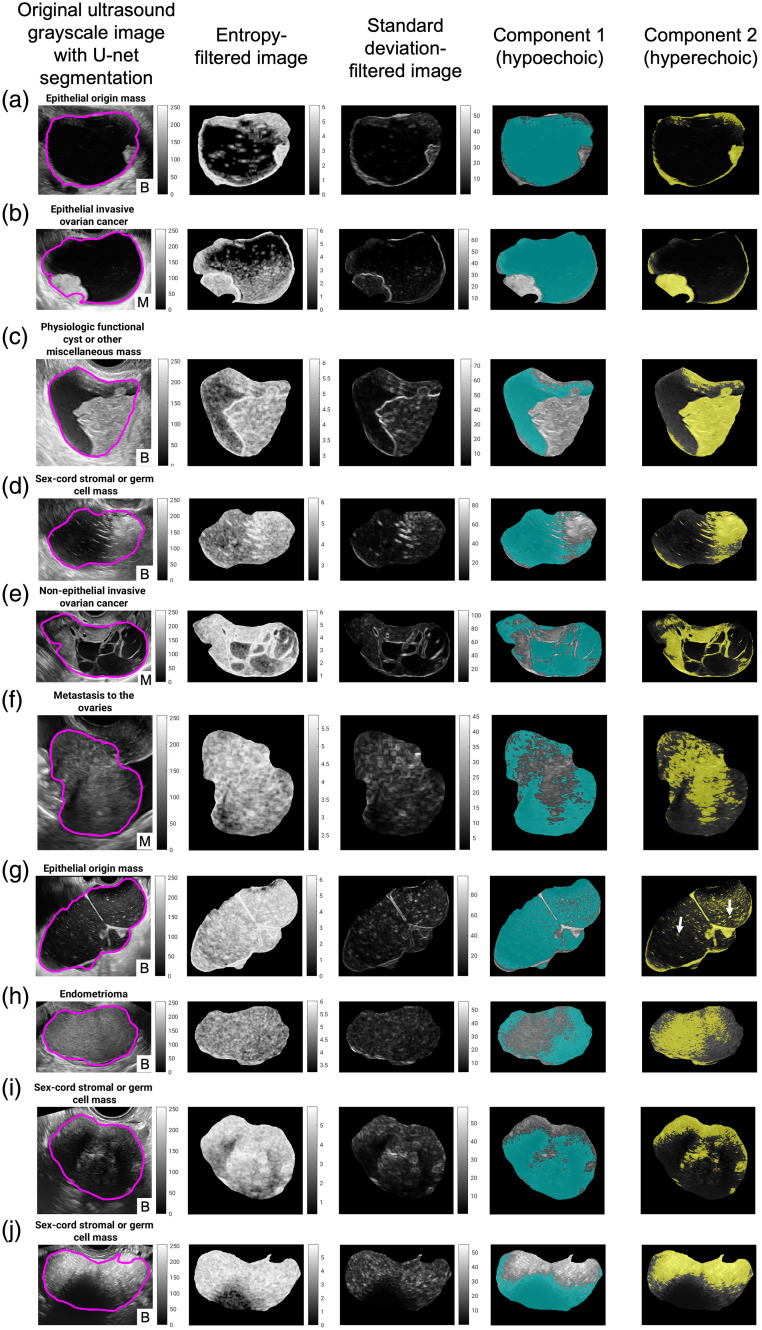
(a)–(j) Example results of internal component segmentation using an unsupervised FCM algorithm. Mass pathology subtypes are as given in Table 1 (i.e., the pathology subtypes used for dataset splitting). Specific patient diagnoses for these masses are described further in the text. B, benign and M, malignant.

The components of the masses from the unsupervised FCM algorithm largely corresponded with the underlying mass characteristics in terms of echogenicity (Fig. 5). Most cystic and solid components in both benign and malignant mass pathology were correctly separated as hypoechoic and hyperechoic respectively, for example as seen in a benign epithelial mass [a cystadenofibroma, Fig. 5(a)] and an epithelial ovarian cancer [malignant clear cell carcinoma, Fig. 5(b)]. Note that retracted blood clots in a hemorrhagic cyst were correctly separated as a hyperechoic component [Fig. 5(c)]. Similarly, the hyperechoic area seen in a germ cell mass [a benign teratoma, Fig. 5(d)] was correctly separated from the hypoechoic area. Additional interesting results were the high-quality detection of intra-mass septations, such as seen in a sex cord-stromal mass [malignant granulosa cell tumor, Fig. 5(e)] as well as the depiction of slight differences in the echogenicity of solid components as seen in ovarian metastasis of gastric carcinoma [Fig. 5(f)].

Different cystic content and echogenicity may influence the clustering of the pixels. For example, mucinous cystadenoma, a benign epithelial mass, that contains mucin gelatinous material with heterogeneous viscosity, often appear on ultrasound as a cystic lesion with low-level internal echoes.^37^ When using the FCM algorithm for two components, some of the mucin particles were labeled as belonging to the hyperechoic component along with the edges of the mass [Fig. 5(g), two example collections of mucin particles indicated with white arrows]. Comparably, cystic lesions containing blood products, such as in an endometrioma, also displayed components consistent with separation in the aged blood accumulation [Fig. 5(h)]. Lastly, we observed that in some cases [e.g., benign ovarian fibroma, Fig. 5(i) and benign teratoma, Fig. 5(j)] acoustic shadowing, which is usually correlated with benign pathologies,^38^ was identified as hypoechoic. We anticipate that this differentiation of acoustic shadowing from other aspects of the masses will enhance the ability of a radiomics pipeline to distinguish between malignant and benign features.

## 4 Discussion

Adnexal mass assessments are challenging tasks requiring knowledge and expertise. Thus there is a rising interest in developing computer-aided diagnosis tools to ease and improve sonographic evaluations, diagnostic accuracy, and patient outcomes. The first and crucial step in a machine learning pipeline is an accurate outlining of the region of interest, the adnexal mass borders. The study results show that using a U-net architecture for automatic segmentation of adnexal masses on ultrasound images has excellent agreement with expert manual outlines, with a Dice score of 0.91 and $R_{\mathrm{HD}\text{-}D}$ of 0.04. This approach has the potential to improve operational efficiency and clinical workflow as it only requires the clinicians to define a bounding box surrounding the adnexal mass, thus also reducing the variability in manually outlining the region of interest. Several groups have studied the use of the U-Net architecture or its variations for segmentation of areas of interest on medical images across a variety of anatomies and physiologies as well as modalities,^39^^–^^42^ including ultrasound,^43^ computed tomography,^44^^,^^45^ and magnetic resonance imaging^46^ of ovarian masses. However, the use of U-net segmentation for adnexal mass assessments on ultrasound has been limited. In addition, most ultrasound-based radiomics classification studies for adnexal masses have used manual segmentation.^47^^–^^52^ One recent study examined the reproducibility of radiomics features extracted from ultrasound images after U-net-based segmentations from 127 patients diagnosed with ovarian cancer.^53^ Although they evaluated different variations of the U-Net algorithm, resulting in mean Dice scores between 0.81 and 0.87 compared to the expert outlines, their segmentation of benign adnexal masses was not assessed, and classification performance for the prediction of benign versus malignant masses was not reported. Recently, Barcroft et al.^54^ reported a segmentation and classification approach for adnexal masses on ultrasound; they evaluated several DL architectures for the segmentation task, and their best-reported U-net model resulted in a median Dice score of 0.85 in an external test set (184 masses) compared to manual expert outlines.

Unsupervised FCM clustering automatically identified clinically-relevant internal mass components, which we hypothesize to be important for the future differentiation of these heterogeneous mass aspects via feature extraction and merging. By our implementation design, the FCM algorithm did not differentiate between types of hyperechoic components, i.e., between solid elements, solid-appearing, and mimickers at the same FCM level.^10^^,^^14^^,^^55^ Future work will explore the differences in the radiomic features of the various hypo- and hyper-echoic components by pathology subtype and their effect on malignancy prediction. Additionally, gelatinous material within a mucinous cystadenoma was hyperechoic, along with the rim of the mass. This may point to the need to differentiate the hyperechoic component along the edges of the mass from any presence of hyperechoic material inside the mass when conducting future feature extraction.

To our knowledge, this study is the first to combine a supervised U-net segmentation approach with an unsupervised FCM approach for the segmentation of mass components for both benign and malignant masses prior to feature extraction and AI classification tasks. Chiappa et al.^49^ studied the importance of a separate feature analysis by the tissue constituent in ovarian masses from 241 patients. They developed three classification systems based on mass appearance: cystic, solid, or mixed. However, in that study, the masses were outlined manually, while our approach is fully automated and requires no labels for the internal components. Lebbos et al.^26^ also used a U-net model for the segmentation of ovarian masses from 222 patients, along with synthetic images, while additionally identifying the internal components as cyst locules, solid elements, or papillary projections based upon manual labels.

Our study has some limitations, typical for an investigation of this nature. First, the study was limited to a segmentation task (i.e., segmentation of adnexal masses from the background, followed by internal component segmentation). By design, it did not incorporate a mass detection task or mass classification task, as we are especially interested in segmenting masses based upon user-provided ROIs around the masses, and we are currently engaging in an in-depth study of component-based radiomic feature extraction and merging for mass classification. Second, it was a single-center retrospective study. Future work will apply the model to images acquired at other institutions, pivotal for generalizability. Third, our dataset represented the inclusion of only adnexal masses that fit specific criteria, with exclusions applied at the patient, mass, and image levels. Ultrasound images of a given pelvic mass can vary by the equipment manufacturer, sonographer expertise and preferences, image acquisition factors such as transvaginal versus transabdominal approach, and image processing parameters including but not limited to depth and gain. Ultrasound is also affected by sound wave traits in biological tissue, which can result in speckle noise, artifacts, and acoustic shadowing. All of these factors impact any AI pipeline for ultrasound in medical imaging.^56^ In this study, we sought to control the impact of these factors through stringent exclusion criteria. Future steps will address these limitations by (a) investigating the application of the segmentation and future classification model to masses that were conservatively managed (i.e., did not progress to surgery); (b) incorporating additional pelvic mass etiologies; (c) studying the impact of user variability and training on segmentation performance (including at the level of the sonographer’s choice of images to archive for the patient); (d) including other image acquisition modes, such as Doppler-based acquisition; and (e) expanding the segmentation task to incorporate edge detection at the edge of the ultrasound image when relevant. The latter, in particular, will expand the model’s applicability to real-world scenarios in which complete visibility of the lesion edge is not always possible. Fourth, we did not study other variations of U-net algorithms or investigate the impact of user variability in ROI selection. These will be our focus in the near future.

## 5 Conclusion

Using a U-net algorithm to automatically outline adnexal masses from a bounding box had an excellent agreement with expert outlines based on performance metrics of DSC and $R_{\mathrm{HD}\text{-}D}$. Furthermore, an unsupervised, automated approach for segmenting internal mass components correlated well with the clinical review of the components. Future work will apply the combined U-net and FCM methods to a larger dataset and investigate radiomic feature-based ultrasound classification of adnexal masses as malignant or benign, potentially providing a comprehensive and simple physician’s decision-support tool that can improve the differentiation between benign and malignant adnexal masses.

## Data Availability

The data used for this article, including ultrasound images, are not publicly available due to patient privacy and data sharing agreements.
